# The European Upper Palaeolithic Palaeoecological and Archaeological Dataset for sites north of 50°N

**DOI:** 10.1038/s41597-026-07586-5

**Published:** 2026-06-05

**Authors:** Tilman Böckenförde

**Affiliations:** CCEHN – Seminar of Prehistory and Early History, Georg-August-University Göttingen, Göttingen, Germany

## Abstract

The European Upper Palaeolithic Palaeoecological and Archaeological Dataset for sites north of 50°N (EUPPAD) presents a systematic collection of palaeoecological and archaeological data from Upper Palaeolithic sites across Europe north of 50°N. Spanning c. 47,000–14,000 cal BP, this dataset integrates site, lithic assemblages, faunal, floral, and contextual records to support meta-analyses of human-environment interactions during pronounced climate fluctuations. EUPPAD covers eight major Upper Palaeolithic technocomplexes, including the Lincombian-Ranisian-Jerzmanowician, Aurignacian, Gravettian, Epigravettian, LGM-sites, Magdalenian, Hamburgian, and Creswellian. Data were compiled from an extensive literature review and published datasets, complemented by field records. Each entry includes detailed contextual, artefactual, and palaeoecological variables, supporting diverse and nuanced research on settlement patterns, resource exploitation, and adaptation to Greenland Interstadials and Stadials. FAIR data principles ensure open access and interoperability. EUPPAD is a comprehensive resource fostering interdisciplinary research into Late Pleistocene climate as well as ecological variability and human adaptation.

## Background & Summary

### The 50th parallel north: A distinctive climatic and archaeological frontier

Late Pleistocene Europe north of 50°N stands at the climatic frontier. Its proximity to the southern margins of the Fennoscandian and British–Irish ice sheets^1,2^ meant direct exposure to periglacial processes, including glacial advances and retreats^3,4^, the catastrophic drainage and flooding of proglacial lakes^2,5^, sea-level changes^6–8^, and severe permafrost^9–11^. The geographic mosaic north of 50°N during the Upper Palaeolithic comprised a mix of lowland plains, low mountain ranges, and coastal landscapes, each responding differently to environmental drivers^12^. The landscape alternated between dry, windblown desert-steppe during cold phases^13^ and periodically inundated lowlands, as seen with the expansion and later flooding of Doggerland^3,7^. Doggerland once connected Britain and continental Europe, persisting as dry land through several stadial and interstadial phases (notably GS-2.1, GI-1e, GI-1c, and GS-1)^14,15^, with profound implications for mobility and resource distribution. The north thus acted as an early indicator for climatic change, with ecological shifts and population responses often preceding those in more temperate regions. As an ecologically sensitive zone, it was particularly vulnerable to even small climate oscillations^16^, resulting in pronounced population fluctuations, repeated episodes of abandonment and recolonisation^17,18^, and strong adaptation pressure on both human^19–21^ and animal communities^22^.

During stadial phases, the dominance of steppe-tundra^23^, the prevalence of permafrost and the scarcity of woody vegetation^13,24^ limited resource diversity for humans and fauna^20,25,26^ alike. Interstadials brought episodes of climatic amelioration, supporting a broader spectrum of vegetation^13,24,27^, and allowing for more sustained – and sometimes higher abundances of fauna^28^. These environmental windows enabled both the pioneering of new areas and intensified interaction between populations and different ecological habitats like lowlands, uplands, and coastal regions. The post-LGM warming and deglaciation^1^, culminating in the Meiendorf Interstadial^29^ (from c. 14,800 cal BP), transformed the northern landscape through rapid reforestation, rising sea levels, and the inundation of former landmasses such as Doggerland^14,15^. These changes underpinned a new phase of human expansion and diversity, as well as increasingly complex adaptations to an evolving environment.

### Background to palaeoecological data use in archaeology

Palaeoecological data have played a pivotal role in Palaeolithic research since the discipline’s early days^30^. Initially, environmental reconstructions relied heavily on interpreting faunal remains, which were often merely categorised into ‘cold-adapted’ or ‘warm-adapted’ species and used as rough chronological markers or simplistic indicators of human presence. However, advances in analytical methods and theoretical approaches now allow for a significantly more detailed and nuanced integration of palaeoecological proxies into archaeological research.

Today, palaeoecological evidence in archaeology encompasses a diverse range of biological, geological, and geochemical datasets derived from archaeological sites and their surrounding contexts. Key proxies include pollen, plant macrofossils, charcoal, non-pollen palynomorphs^13,27,31^, and sedimentary ancient DNA (sedaDNA)^32–36^, alongside the traditional study of faunal assemblages. These proxies, often combined with improved radiometric dating and stable isotope analyses, offer multi-scalar reconstructions of climate, vegetation, and biotic communities over time^24,37,38^.

In Upper Palaeolithic research, the integration of these datasets is crucial for understanding human adaptation to rapid and pronounced climatic oscillations, such as those of the Greenland Interstadials and Stadials^39–48^. Pollen and plant macrofossil analyses provide detailed records of vegetational change, indicating shifts between open steppe-tundra and (open-)forest environments that would have affected resource availability for hunter-gatherer populations^13,24,27,31,49^. Faunal analysis has evolved from simple dichotomies to complex studies of population dynamics, extinction events^49–57^, and the biogeography of prey species^58^ with respect to both climate and human activity^28,59–61^.

Moreover, stable isotope analysis of both human and animal remains yields insights into palaeodiet, habitat preference, and mobility strategies^20,62–69^. Ancient DNA techniques begin providing an unprecedented level of detail regarding past biodiversity, species dispersal, and ecosystem resilience. Rather than acting as broad environmental backdrops, such palaeoecological records are now fundamental in testing archaeological hypotheses concerning human responses to environmental change^70^, patterns of migration^71^, and the drivers of cultural and technological innovation and adaptation^21,72,73^.

### Introducing the EUPPAD dataset

Developed within the framework of the ‘Climate Change and Early Humans in the North (CCEHN)’ project (https://ccehn.de/), the European Upper Palaeolithic Palaeoecological and Archaeological Dataset (EUPPAD) has been developed to provide a comprehensive and systematic collection of the archaeological and palaeoecological record of Upper Palaeolithic sites located north of 50°N. As the integration of palaeoecological data becomes increasingly essential for understanding the contexts, adaptations, and behaviours of Palaeolithic populations, the need for a centralised and standardised dataset has grown correspondingly. EUPPAD is designed to address this requirement by collating a wide range of faunal, floral, artefactual (lithic and organic), and contextual site data, thereby enabling nuanced analyses of human–environment interactions during this period of dynamic climatic and environmental changes in prehistory.

The dataset adheres to the principles of FAIR data management – ensuring that information is Findable, Accessible, Interoperable, and Reusable. By making these extensive records openly available, EUPPAD aims to serve as a valuable resource supporting reproducible research and the testing of new hypotheses concerning Upper Palaeolithic lifeways, site use, resource exploitation, and responses to past climate change. Moreover, the dataset facilitates large-scale comparative studies and cross-regional syntheses, promoting collaborative palaeolithic research on a continental scale.

## Methods

### Data recording

All data, site chronologies, and associated archaeological technocomplexes reported herein pertain exclusively to regions situated north of 50°N, in accordance with the geographic and chronological parameters of the present dataset. In general, only publicly available sources were used in the construction of EUPPAD. The majority of site information was obtained through an extensive literature review, while site data for Abri Stendel XVIII, Friedrichsdorf-Seulberg^74^, Hachborn 35^75^, and Dreieich-Götzenhain Nord and Ost^76^ was derived from excavation or survey records compiled by the author. To obtain an initial overview of the sites and technocomplexes, several published datasets were consulted^77^, and some artefact^78^ and microfauna-related data were incorporated^79^.

### Chronological selection

**Fig. 1:**
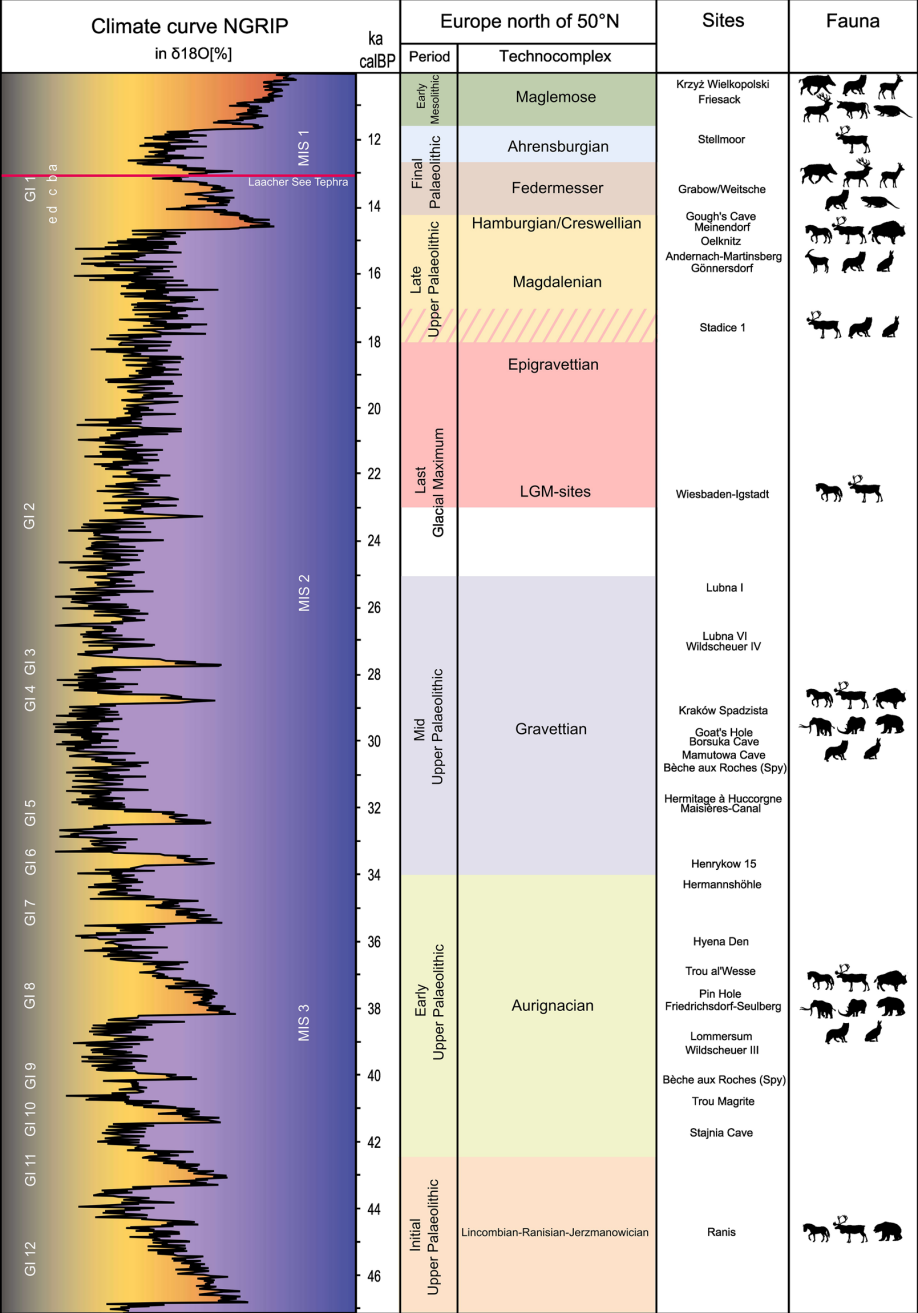
Schematic figure of the Upper Palaeolithic technocomplexes north of 50°N, selected radiocarbon dated sites, documented fauna against the NGRIP curve.

**Fig. 2:**
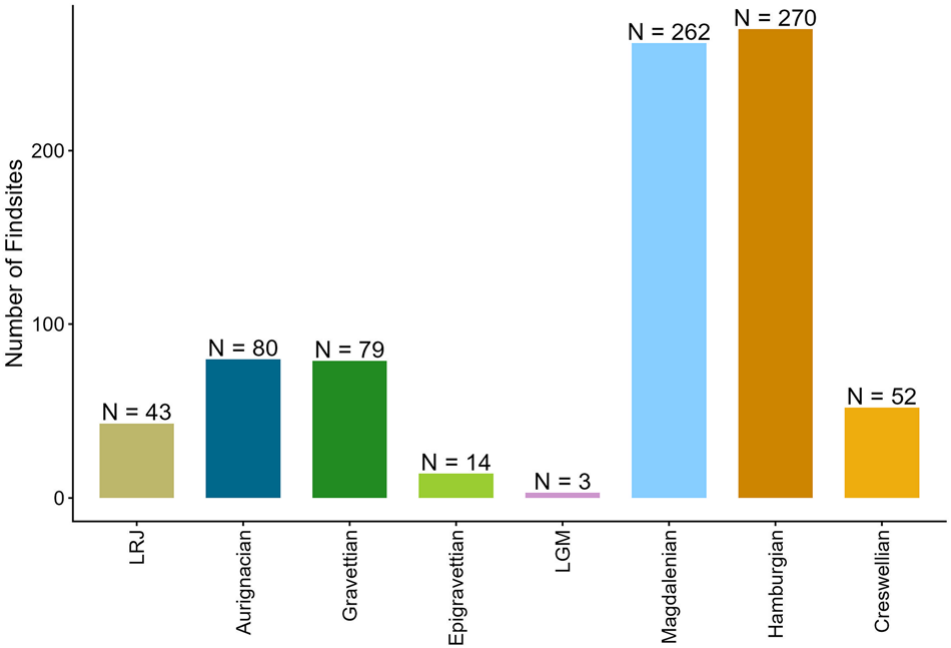
Distribution of Upper Palaeolithic find sites in the dataset per technocomplex north of 50°N (N = 803).

Chronologically, the dataset covers sites attributed to eight major Upper Palaeolithic technocomplexes connected to *Homo sapiens* (Fig. 1), from the Early Upper Palaeolithic Lincombian-Ranisian-Jerzmanowician (LRJ) to the Late Upper Palaeolithic Hamburgian and Creswellian (Fig. 2). For all technocomplexes except the Hamburgian and the Creswellian, the 50°N serves as a practical demarcation line, representing the northernmost extent of their known distributions.

**Fig. 3:**
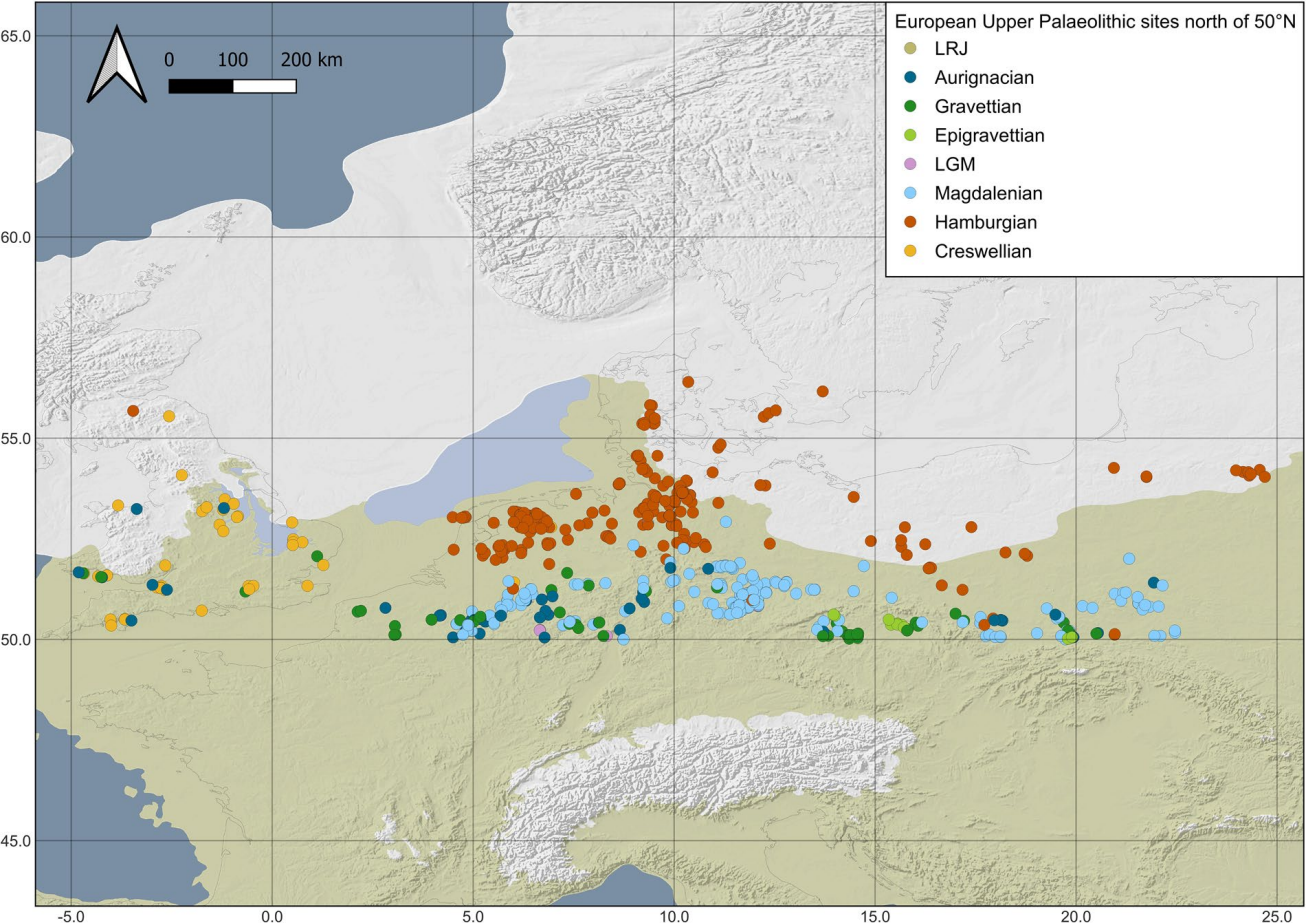
Distribution of find sites in the dataset north of 50°N. The background map displays the maximum extension of the ice sheets during the LGM. Map compiled by ZBSA^1,2,5,6,8,109–121^.

The LRJ (c. 47,000–43,000 cal BP^80–83^) populations expanded into northern Central Europe (Fig. 3) roughly during the relatively temperate climates of GI 13–11 and GS 13-11^43^ (Fig. 1). Generally, the LRJ is seen as part of the Initial Upper Palaeolithic (IUP) with partial-bifacial blade points of the Jerzmanowice type and bifacial leaf points, but north of 50°N, all sites connected to the IUP are exclusively represented by the LRJ, whereof 75% are located in the UK. The Aurignacian (c. 42,000–34,000 cal BP^84–87^) corresponds with roughly GI 11–6 and GS 11–6^43^ (Fig. 1), developing amidst growing climatic instability and the spread of periglacial conditions.

The Gravettian (c. 34,000–25,000 cal BP^70,85,88–92^), spanning c. GI 6–3 and GS 6–3^43^ (Fig. 1), coincided with ongoing cold phases and the approach of the Last Glacial Maximum (LGM). The Epigravettian (c. 23,000–18,000 cal BP^93–95^) persisted through c. GI 2.2–1 and GS 2.2–2.1a^43^ (Fig. 1), maintaining broad-spectrum subsistence strategies in southern and eastern refugia^64,93,96^. North of 50°N, Epigravettian evidence is sparse and limited to (south)eastern Europe (Fig. 3). The LGM (c. 25,000–20,000 cal BP^97,98^) is roughly aligned with GI 2.2 and the coldest phase of GS 2.2–2.1c–b^43^ (Fig. 1). For this, only western European sites are considered LGM-sites (Fig. 3), even though some Epigravettian also fall within the ‘climatic’ LGM. Notably, this definition considers isolated sites, e.g., Wiesbaden-Igstadt^98,99^ and Gera-Zoitzberg^97^, that by typological standards would not be considered Epigravettian or Badegoulian. The Magdalenian (c. 18,500–14,800 cal BP^78,100^) is associated with GS 2.1^43^ (Fig. 1), reflecting relatively stable, but persistently cold and arid stadial environments. The Hamburgian and the Creswellian (c. 14,800–14,000 cal BP^101,102^) matches the Meiendorf Interstadial (GI 1e^43,101,103^, Fig. 1), marking a significant warming event. The Hamburgian technocomplex is primarily situated within the North European Plains whereas the Creswellian is mostly documented in the UK (Fig. 3). Thus, all identified Hamburgian and Creswellian sites within this region have been included in the dataset.

Chronological attributions of sites were adopted directly from published sources and omitted only in instances of dubious or contested identification (e.g., Kremmen). The aim of the dataset is not to interpret the temporal attribution of sites, but rather to collate published data for future research use. However, a substantial number of surface collections are compiled in this dataset, and their chronological attribution often leaves room to scrutiny since it was mostly done by researchers on diagnostic lithic artefacts, often many decades ago.

### Site selection geographical distribution

EUPPAD comprises a total of 803 sites, spanning from the British Isles in the northwest to the Baltic in the northeast, and encompassing all European Upper Palaeolithic sites located north of 50°N (Fig. 3). This geographical extent includes present-day United Kingdom, Northern France, the Netherlands, Belgium, Northern Germany, Denmark, Poland, Southern Sweden, Lithuania and Northern Czech Republic. The northern part of the study area is dominated by the North European Plain, while its southern boundary coincides with the low mountain ranges that closely follow the 50°N.

#### Typological parameters and raw materials

Lithic artefacts were categorised as blanks (n = 5) or tools (n = 22) and systematically recorded. On rare occasions, particularly in older publications, artefact attributions were updated when clear misidentification was evident. In general, artefact typology was taken from the literature. All types of burins were documented as burins since burin typology changed significantly over palaeolithic research history, and reclassification could not be ensured safely. Additionally, the number of bone points was also collated. The total number of artefacts was also taken as a variable, but added as the sum of all recorded artefacts.

In addition, the documented lithic raw materials (n = 52) of all sites were recorded in the dataset. This was undertaken to enable future mobility reconstruction by researchers.

### Palaeoecological parameters

**Fig. 4:**
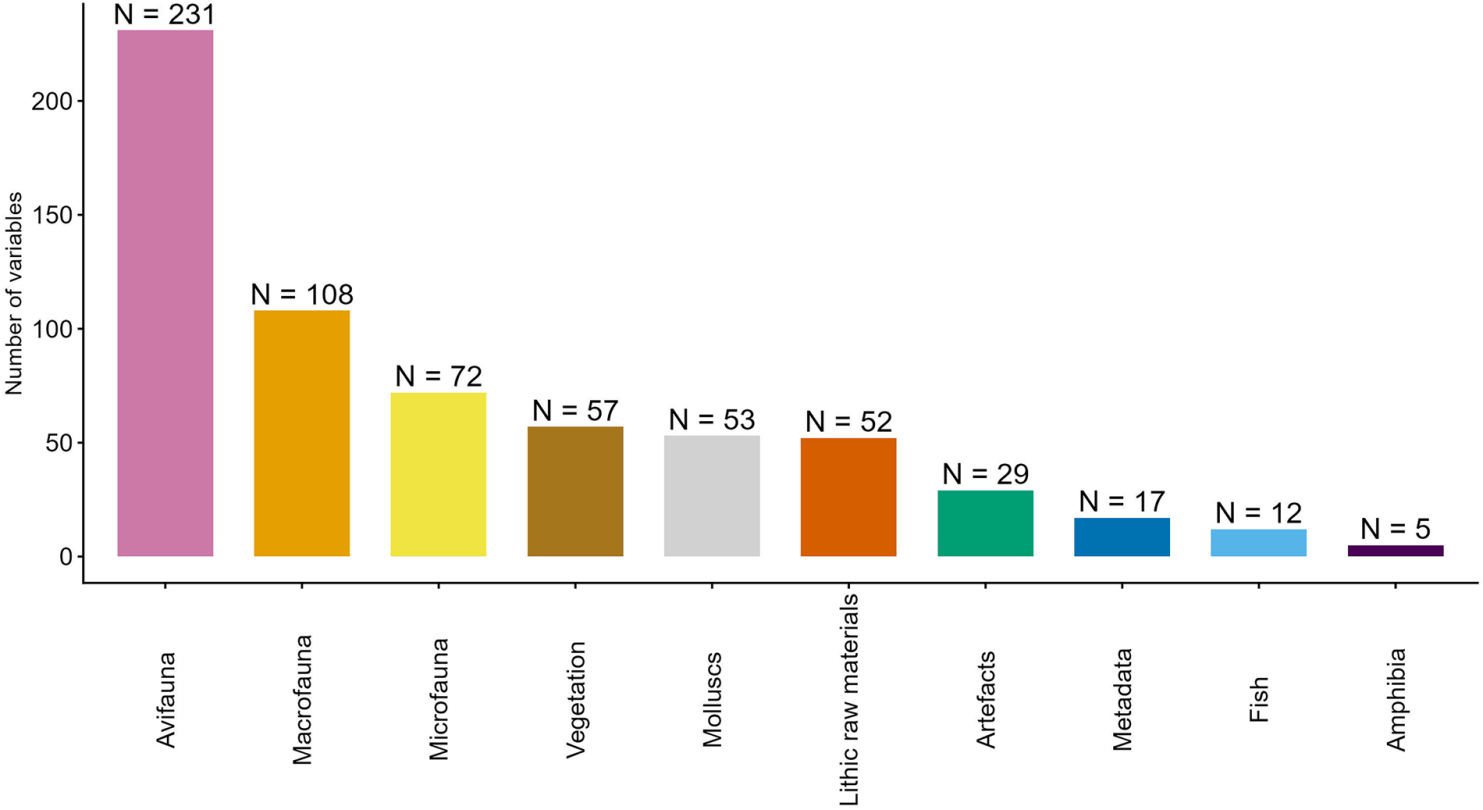
Overview of the collected number of variables per category (N = 636).

A total of 538 palaeoecological variables were recorded from the literature for each location (Fig. 4). These variables encompass faunal and vegetation records. Faunal and vegetation data were collected at the taxonomic level (whenever possible) or otherwise at the genus level. Faunal remains were further subdivided into macrofauna (>2 kg), microfauna (<2 kg), avifauna (birds), fish, molluscs, and amphibians. For megafauna, separation between bones, teeth and ivory (*Mammuthus primigenius*) has been made if information had been available. For all taxa the number of individual specimen (NISP) and minimum number of individuals (MNI) was recorded (if available). Vegetation evidence was classified according to pollen or charcoal and recorded by taxon wherever the literature permitted.

### Radiocarbon data

579 radiocarbon dates for 123 sites were collected by literature review and/or the Radiocarbon Palaeolithic Europe Database v29^104^ and PACEA^105^ or obtained by the author (e.g., Abri Stendel XVIII, Friedrichsdorf-Seulberg). Data were then cross-validated against primary publications and secondary literature for each site whenever possible. As many published dates have to be regarded as unlikely or dubious, rigorous data hygiene was conducted following the protocol of Pedersen *et al*.^102^, excluding all dates not taken from modified faunal remains or from insecure stratigraphy. In an additional table (‘*euppad_radiocarbon_dates_selected*’), if several plausible dates for one site were available, the dates with the lowest standard deviation (in most cases the newest due to advanced pre-treatment processes) were retained. In order to better indicate variable confidence in all collated sites, a site-ranking based on radiocarbon dating and excavation evidence was created (see Data validation).

### Limitations of the data

As previously mentioned, a large quantity of sites is represented by surface collections that were not excavated. These sites add quantitative value to any spatial distribution analyses but lack any real qualitative depths that could be exploited for further detailed statistical analysis. Also, their chronological assessment is based on a few diagnostic artefacts and may change if re-analysed or excavated. Further, given the long and extensive palaeolithic research history in Europe, some sites excavated decades ago lack qualitative data based on the excavation technique and documentation standards used at that time (e.g., cave sites in the UK, Belgium and Poland). The dataset also includes information on lost assemblages (e.g., Rhens), which makes any validation of the data impossible. Given the distribution of soils not ideal for organic preservation, specifically in the Northern European Plains, some sites in the dataset (e.g., Hamburgian) lack organic preservation and therefore the available data does not reflect their respective palaeoecological conditions.

## Data Records

### Folder structure

EUPPAD^106^ is available on the open-access repository Zenodo under this link: https://zenodo.org/records/19930467. This includes a CSV file including all data called ‘*euppad_complete’*, as well as separated CSV-files for all categories (‘*euppad_metadata*’, ‘*euppad _artefacts*’, ‘*euppad _lithic_raw_materials*’, ‘*euppad_macrofauna*’, ‘*euppad_microfauna*’, ‘*euppad_avifauna*’, ‘*euppad_fish*’, ‘*euppad_molluscs_and_other*’, ‘*euppad_amphibia*’, ‘*euppad_charcoal_pollen*’). Radiocarbon dates are presented in two separate CSV file called ‘*euppad_radiocarbon_dates_all*’ and ‘*euppad_radiocarbon_dates_selected*’. All files include the EUPPAD_id and site name to cross-link between tables and to ensure data transfer to secondary platforms such as R or GIS. The bibliography of the EUPPAD dataset on Zotero is available under this link: https://www.zotero.org/groups/6251724/the_euppad_database; the corresponding Word file ‘*euppad_references*’ is also on Zenodo. Literature up to March 2026 was considered during dataset compilation. All records have been systematically organised in a PostgreSQL dataset. Table 1 provides an overview of all categories used in the creation of the dataset.

**Table 1:** Overview of the metadata categories in the EUPPAD dataset.

Category	Legend
EUPPAD_id	Dating_country code_ID (LRJ = Lincombian-Ranisian-Jerzmanowician, AU = Aurignacian, GR = Gravettian, EPI = Epigravettian, LGM = Last Glacial Maximum, MA = Magdalenian, HA = Hamburgian, CRE = Creswellian)
site	Name of the site
lon	Coordinates longitude
lat	Coordinates latitude
accuracy	1 = precise, 2 = near, 3 = periphery
country	Country (BE = Belgium, DK = Denmark, GE = Germany, FR = France, UK = United Kingdom, LU = Luxemburg, NL = Netherlands, PL = Poland, CZ = Czech Republic, LI = Lithuania, SW = Sweden)
state	State
municipality	Municipality
dating	LRJ, Aurignacian, Gravettian, Epigravettian, LGM, Magdalenian, Hamburgian, Creswellian
site_type	1 = Cave, 2 = Abri, 3 = Open-Air Site
excavation	No: date if available; TRUE = Yes, date unclear; FALSE = No
site_ranking	A, B, C, D, E
soil_type	Categorisation by type of soil (e.g., sand, clay, silt).
ams	AMS-dating (TRUE = Yes; FALSE = No)
conv 14c	Conventional ^14^C-dating (TRUE = Yes; FALSE = No)
references	References

### Metadata

**Fig. 5:**
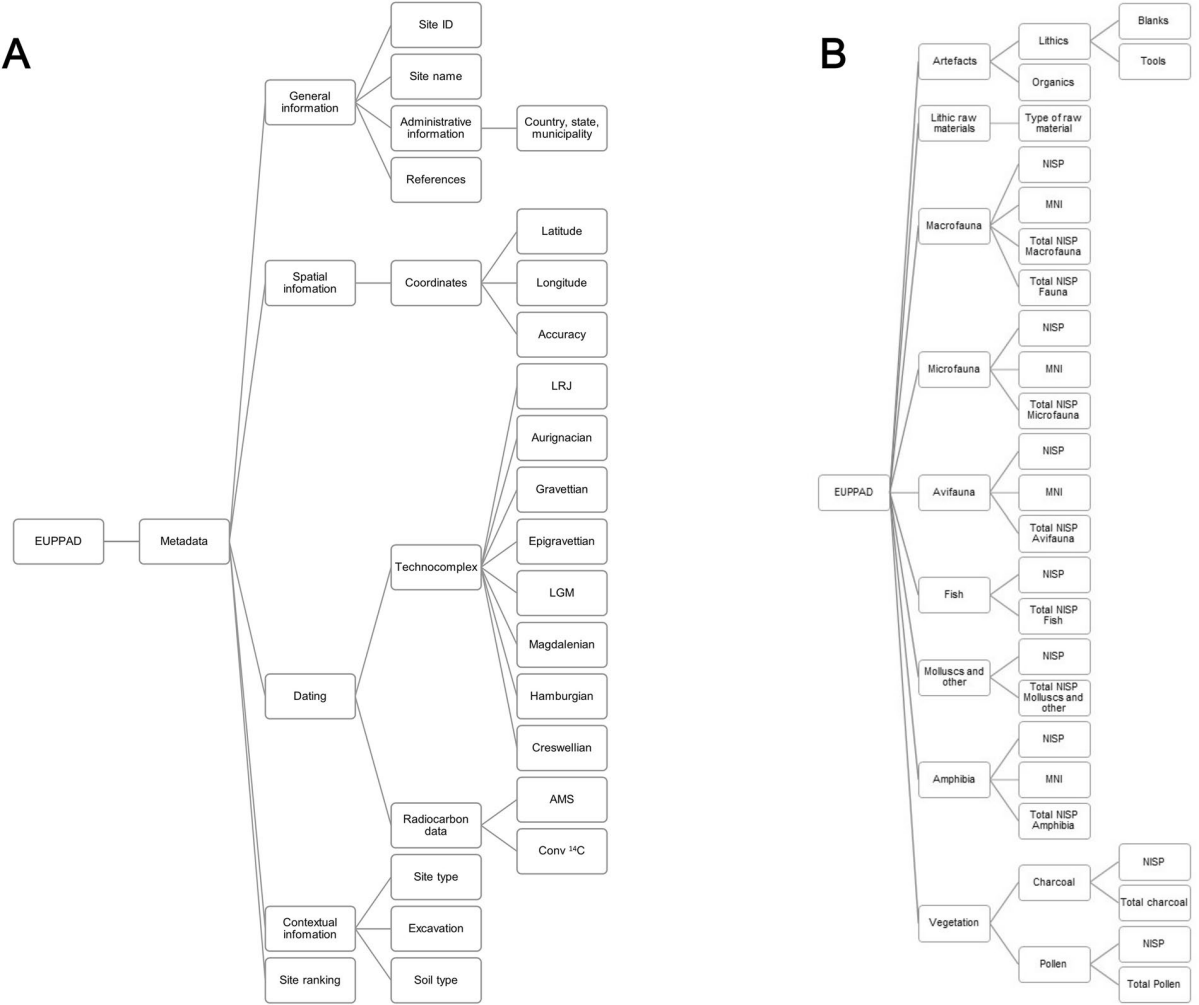
Schematic hierarchical view of the EUPPAD dataset and its parameters. (**A**) Metadata and its subdivisions. (**B**) Artefactual, lithic raw materials and palaeoecological parameters and its subdivisions.

The metadata (Fig. 5, Table 1) spans general information such as EUPPAD_id, site name, administrative information and references. Spatial information contains WGS 84 geographical coordinates (lon, lat) and coordinate accuracy (1 = precise, 2 = near, 3 = periphery). Chronological attribution contains typological (technocomplexes) and radiocarbon dating (AMS and conventional ^14^C). In the metadata table, the availability of radiocarbon dates for each site is given in *TRUE* or *FALSE*. Metadata also entails some contextual information, such as soil type (e.g., sand, loess, etc.), excavation dates if available, and site type (1 = cave, 2 = abri, 3 = open-air site). Binary *TRUE* or *FALSE* are given if information is available but lacks exact details or is negative. *NA* is given if information is generally not available in the sources.

### Handling of typological and ecological parameters

Where the precise number of artefacts could not be determined from published reports, binary *TRUE* or *NA* values were assigned (Table 2), notably for surface collections, to ensure clarity and prevent the mishandling of data. *TRUE* represents presence data of unknown quantity. *NA* represents unavailable information at both excavated and not excavated sites. *FALSE* was only added for lithic raw materials at excavated sites and represents absence data.

**Table 2:** Overview of the palaeoecological and archaeological parameters in the EUPPAD dataset.

Category	Legend
first column fauna taxa_BO	No. of identified specimen – bones (NISP) (No = Exact Number; NA = No information available; TRUE = Present but number unclear; TIME = Not present because of time constraints)
second column fauna taxa_TH	No. of identified specimen – teeth (NISP) (No = Exact Number; NA = No information available; TRUE = Present but number unclear; TIME = Not present because of time constraints)
third column fauna taxa_IV	No. of identified specimens – ivory (NISP) (No = Exact Number; NA = No information available; TRUE = Present but number unclear; TIME = Not present because of time constraints)
fauna_taxa_MNI	No. of min. individuals (MNI) (No = Exact Number; NA = No information available; TRUE = Present but number unclear)
faunal_remains_total	No. of total faunal remains across all categories
category_total	No. of total faunal remains in each category
charcoal	No. of charcoal specimen (No = Exact Number; NA = No information available; TRUE = Present but number unclear)
pollen_total	No. of total pollen specimens (No = Exact Number; NA = No information available; TRUE = Present but number unclear)
first column pollen taxa	No. of pollen specimen (No = Exact Number; NA = No information available; TRUE = Present but number unclear
raw_material	Categorisation by type of raw material (e.g., chert, jasper, quartzite, limestone). TRUE = Present; FALSE = Absent, NA = No information available
artefacts_total	No. of total artefacts across all artefact types
artefacts_type	Categorisation by type of artefact (blanks, tools, organics).

For all flora and fauna parameters, the number of the observed specimen, *TRUE* for presence data and *NA* for absence data was added. *FALSE* was not added for fauna or flora since it could not be ruled out that certain taxa were present at a given site, but were not preserved. At some sites, faunal remains were documented belonging to – at that time – extinct species. Therefore, in certain technocomplexes (e.g., *Ursus spelaeus* in the Hamburgian), *TIME* for recorded for extinct species as an indicator that this species could not belong to the archaeological finds and may be an older relict. However, given the secondary use of faunal remains (e.g., pre-LGM dated mammoth bone in Gönnersdorf), there are cases where *TRUE* has been added for a species for a certain site even though that species may have been extinct had that time. In addition, some sites exhibit taxa that may not have been able to exist during the actual occupation time of the site (e.g., beaver at Bettenroder Berg IX^107^ or certain species of bird at Belgian sites^108^). However, due to the possibility of non-analogous faunal assemblages the author chose to keep these taxa and leave the final decision of in- or excluding specific data to the end user.

#### Handling of radiocarbon dates

To ensure the data quality of all radiocarbon dates, primary sources were checked and several parameters for each date collected in the table (Table 3). Great care was taken to only include dates that derive from anthropogenically modified faunal remains or charcoal that derives from hearths or other anthropogenic features. In some cases, information on the feature was not available, then *NA* was given. The primary source has always been stated in ‘references’.

**Table 3:** Overview of categories of radiocarbon dates in EUPPAD.

Category	Legend
EUPPAD_id	Dating_country code_ID (LRJ = Lincombian-Ranisian-Jerzmanowician, AU = Aurignacian, GR = Gravettian, EPI = Epigravettian, LGM = Last Glacial Maximum, MA = Magdalenian, HA = Hamburgian, CRE = Creswellian)
site	Name of the site
lon	Coordinates longitude
lat	Coordinates latitude
method	AMS or Conv ^14^C
labnr	Laboratory number of radiocarbon date
c14age	BP-age of radiocarbon date
c14std	Standard deviation of radiocarbon date
feature	Origin of the sample
material	Organic material of the sample
references	References

## Technical Validation

Every effort was made to ensure the integrity of the dataset; however, the sites included have research histories spanning over 150 years. Consequently, the quality of documentation and publication for artefacts and palaeoecological data varies considerably.

To distinguish sites according to the reliability of their documentation, a site ranking index based on ^14^C-dates as well as excavations conducted was introduced: sites ranked A are considered the most reliable, while sites ranked E denote surface finds with less secure contextual information (Table 4). As this dataset aims to quantify human occupation of sites and not the geological or faunal age of layers, the available radiocarbon data was filtered to include only dates that come from a secure stratigraphy, that derive from modified artefacts and reliable dates due too good collagen quality. It is recommended that this ranking be utilised when selecting sites for further analytical modelling to account for potential disparities in data quality across the dataset. The ‘*euppad_radiocarbon_dates_selected*’ table was used for the creation of the site ranking.

**Table 4:** Overview of the site ranking categories in EUPPAD.

Category	Excavation	AMS	Conventional ^14^C
A	TRUE	TRUE	TRUE or FALSE
B	TRUE	FALSE	TRUE
C	FALSE	TRUE or FALSE	TRUE or FALSE
D	TRUE	FALSE	FALSE
E	FALSE	FALSE	FALSE

## Usage Notes

### Disclaimer

This Data Descriptor was peer-reviewed in 2026 using the data available on the platform at that time. The future chronological (e.g., addition of the Final Palaeolithic) and spatial expansion (south of 50°N) of this dataset is planned. For the most current information, refer to the latest version available on the platform. The bibliography will continuously be updated on Zotero accordingly.

An R package for easy access as well as fast and convenient data integration is also planned for future updates and will be made available on GitHub.

### Perspective on data usage

A whole range of applications is imaginable using the dataset. This includes – but is not limited to – a range of exploratory and inferential analyses across spatial, temporal, and taxonomic scales, including regression-based approaches (LM/GLM/GLMM) and multivariate ordination to investigate patterns in abundance, occurrence, and community composition. Additionally, the radiocarbon dates could be the basis for Bayesian chrono-modelling on a cultural or spatial level.

## Data Availability

EUPPAD is available on the open-access repository Zenodo under this link: https://zenodo.org/records/19930467.

## Code availability

No custom code was used in the collection and creation of the data, nor is it required for the use of the dataset.
